# Pharmacotherapy assessment in chronic kidney disease: validation of the PAIR instrument for use in Brazil

**DOI:** 10.1590/2175-8239-JBN-2019-0205

**Published:** 2020-06-01

**Authors:** Alessandra Batista Marquito, Hélady Sanders Pinheiro, Natália Maria da Silva Fernandes, Rogério Baumgratz de Paula

**Affiliations:** 1 Universidade Federal de Juiz de Fora, Faculdade de Medicina, Programa de Pós-Graduação em Saúde, Núcleo Interdisciplinar de Estudos, Pesquisas e Tratamento em Nefrologia, Juiz de Fora, MG, Brasil.; 2 Universidade Federal de Juiz de Fora, Faculdade de Medicina, Departamento de Clínica Médica, Juiz de Fora, MG, Brasil. Núcleo Interdisciplinar de Estudos, Pesquisas e Tratamento em Nefrologia, Juiz de Fora, MG, Brasil.

**Keywords:** Chronic Kidney Disease, Validation Studies, Pharmaceutical care, Doença Renal Crônica, Estudos de Validação, Assistência Farmacêutica

## Abstract

Individuals with chronic kidney disease (CKD) use polypharmacy, which, in combination with renal impairment, exposes them to the risk of drug-related problems (DRPs). There are no available tools in Brazil to systematically assess the pharmacotherapy and management of DRPs in this population. Therefore, the objective of this work was to validate the PAIR instrument (Pharmacotherapy Assessment in Chronic Renal Disease) for use in Brazilian Portuguese. This is a retrospective longitudinal observational study. Medical records from 100 CKD patients under conservative treatment, between 2016 and 2017, in a nephrology clinic, were analyzed. PAIR was applied by pharmacists in two consultations of the same patient, with an interval of 6 months. Reliability, conceptual validity, responsiveness of the instrument and prevalence of DRPs in the studied sample were assessed. A mean of 1.26 ± 0.96 DRPs/patient was identified. Inter-rater reliability coefficients (k) ranged from 0.58 to 0.94 and from 0.79 to 1.00 for test-retest, revealing moderate to perfect level of agreement. In conceptual validity, a mean of 1.60 ± 1.24 DRPs/patient was identified by the nephrologist through clinical judgment, compared to 1.33±0.76 DRPs/patient identified by the pharmacist using PAIR (*p* = 0.07). Therefore PAIR allowed the identification of clinically significant DRPs. In responsiveness, a mean of 1.26 ± 0.96 DRPs/patient was identified at the first consultation and 1.11 ± 1.02 DRPs/patient at the subsequent consultation (*p* = 0.17) by the pharmacist using PAIR. The number of DRPs between the periods did not change. As a conclusion, the PAIR allowed the identification of clinically significant DRPs in CKD, constituting a new validated instrument to be used in Brazil.

## Resumo

Pacientes com doença renal crônica (DRC) utilizam polifarmácia, que, associada ao comprometimento renal, os expõe ao risco de problemas relacionados a medicamentos (PRMs). No Brasil, não existem instrumentos para sistematizar a avaliação da farmacoterapia e a gestão de PRMs nessa população. Portanto, o objetivo deste trabalho foi validar o instrumento PAIR (Pharmacotherapy Assessment in Chronic Renal Disease) para uso em português brasileiro. Trata-se de um estudo observacional longitudinal retrospectivo. Foram analisados prontuários de 100 pacientes com DRC, em tratamento conservador, atendidos entre 2016 e 2017, em clínica de nefrologia. O PAIR foi aplicado por farmacêuticos em duas consultas do mesmo paciente, com intervalo de 6 meses. Avaliou-se confiabilidade, validade conceitual, responsividade do instrumento e prevalência de PRMs na amostra. Uma média de 1,26 ± 0,96 PRM/paciente foi identificada. Na confiabilidade entre avaliadores, o coeficiente k variou de 0,58 a 0,94 e no teste-reteste, de 0,79 a 1,00, revelando grau de concordância moderada a perfeita. Na validade conceitual, uma média de 1,60±1,24 PRM/paciente foi identificada pelo nefrologista, por meio do julgamento clínico, comparado com 1,33 ± 0,76 PRM/paciente identificada pelo farmacêutico, usando o PAIR (*p* = 0,07). Portanto, o PAIR permitiu identificar PRMs clinicamente significativos. Na responsidade, uma média de 1,26 ± 0,96 PRM/paciente foi identificada na primeira consulta e 1,11 ± 1,02 PRM/paciente na consulta subsequente (*p* = 0,17) pelo farmacêutico, usando o PAIR, não sendo observada diferença no número de PRMs entre os períodos. Dessa forma, o PAIR permitiu identificar PRMs clinicamente significativos em pacientes com DRC, constituindo um novo instrumento validado para ser utilizado no Brasil.

## Introduction

Chronic kidney disease (CKD) is a global public health problem, which has a negative impact on the expectation and quality of life of individuals with the disease.^1^^,^ 2 In Brazil, about 133 thousand people undergo renal replacement therapy (RRT), more than twice as much as there was at the beginning of the last century.^3^ The latest national estimates of CKD prevalence and incidence rates in patients undergoing dialysis were 610 and 194 patients per million of the population, respectively.^3^

Among the main risk factors for CKD are diabetes mellitus (DM) and systemic arterial hypertension (SAH), responsible for two thirds of the cases of the disease. This population, made up predominantly of elderly people, has several comorbidities, the approach of which involves the continuous use of multiple medications.^1^^,^ 4 ^,^ 5 Accordingly, data from our outpatient care service for patients with CKD showed the use of polypharmacy by 66, 5% of patients undergoing conservative treatment, on average six drugs per patient, mainly drugs acting on the cardiovascular and metabolic systems.^6^

It is important to note that polypharmacy, combined with renal impairment, which influences the metabolism and excretion of drugs, exposes this population to drug-related problems (DRP).^6^^,^ 7 ^,^ 8

In general, DRP is defined as a health problem related to pharmacotherapy, which interferes or may interfere with the therapeutic outcomes and quality of life of the user,^9^ and can originate during the process of prescription, dispensing or administration of the medication.^10^

In risk populations, such as individuals with congestive heart failure, DM and SAH, DRP are associated with adverse reactions and prescription errors.^11^^,^ 12 In patients with CKD, specifically, there is evidence of a high prevalence of DRP in all stages of the disease,^7^^,^ 13 the most common being the use of contraindicated drugs or in inadequate doses, which can negatively interfere with renal function,^14^ and drug interactions.^6^ The mortality rate associated with the inappropriate use of drugs is 40% higher in patients with an estimated glomerular filtration rate (eGFR) <60 mL/min/1.73 m^2^ compared to patients without CKD.^15^

Given this context, the early detection of DRP in this population may contribute to the prevention of complications and cost reduction in health care through improved survival, reduced disease progression and reduced cardiovascular morbidity.^16^^,^ 17

The Pharmacotherapy Assessment in Chronic Renal Disease (PAIR) instrument was recently developed in Canada, with the purpose of evaluating pharmacotherapy in CKD. PAIR helps with the prevention, detection and management of DPR in individuals with CKD undergoing conservative treatment.

In addition, this is the first and only instrument developed for pharmacists working in nephrology, that allows the identification and management of DRP quickly and systematically, with a focus on medication safety.^10^ For this reason, this clinical support tool has been used as a reference by several authors, in order to guide, standardize and optimize the conduct of these professionals.^18^^,^ 19 ^,^ 20 ^,^ 21 ^,^ 22

Due to the fact that there is no instrument available in Brazil to guide the evaluation of pharmacotherapy, specifically in the population of patients with CKD, our group performed the translation and cross-cultural adaptation of PAIR at an initial phase (Chart 1).^23^

**Chart 1:** PAIR (Pharmacotherapy Assessment in Chronic Renal Disease) Instrument - Version translated and adapted for Brazilian Portuguese23

Drug therapy assessment in chronic kidney disease
**Inadequate use (inadequate dose or contraindicated medication)**
1. The patient is receiving a contraindicated medication, a non-steroidal anti-inflammatory agent.
2. The patient is receiving a very high dose of gabapentin:
GFR 30-59 mL/min: maximum dose of 1,400 mg/day, oral;
GFR 15-29 mL/min: maximum dose of 700 mg/day, oral;
GFR 10-14 mL/min: maximum dose of 300 mg/day, oral;
GFR < 10 mL/min: maximum dose of 150 mg/day, oral.
3. The patient is receiving a contraindicated drug, meperidine.
4. The patient is receiving a very high dose of pregabalin.
GFR 30-59 mL/min: maximum dose of 300 mg/day, oral;
GFR 15-29 mL/min: maximum dose of 150 mg/day, oral;
GFR < 15 mL/min: maximum dose of 75 mg/day, oral.
5. The patient is receiving a very high dose of an antiviral agent (acyclovir, valacyclovir, fancyclovir), according to the dose-adjustments tables for kidney disease.
6. The patient is receiving a very high dose of cephalosporin, according to the dose-adjustment tables for kidney disease.
7. The patient is receiving a very high dose of a neuraminidase inhibitor (oseltamivir, for instance), according to the dose-adjustment tables for kidney disease.
8. The patient is receiving nitrofurantoin, which is contraindicated in kidney disease (GFR < 60 mL/min).
9. The patient is receiving a very high dose of penicillin, according to the dose-adjustment tables for kidney disease.
10. The patient is receiving a very high dose of quinolone, according to the dose-adjustment tables for kidney disease.
11. The patient is receiving a very high dose of sulfonamide, according to the dose-adjustment tables for kidney disease.
12. The patient is receiving a very high dose of tetracycline, according to the dose-adjustment tables for kidney disease.
13. The patient is receiving a very high dose of a triazol (fluconazole, for instance), according to the dose-adjustment tables for kidney disease.
14. The patient is receiving a very high dose of a beta blocker, according to the dose-adjustment tables for kidney disease.
15. The patient is receiving a very high dose of fenofibrate nanocrystals.
GFR 20-50 mL/min: maximum dose of 48 mg/day, oral.
16. The patient has a GFR < 25 mL/min and is receiving a contraindicated medication, acarbose.
17. The patient has a GFR < 30 mL/min and is receiving a contraindicated medication, metformine.
18. The patient is receiving a very high dose of ranitidine.
GFR < 50 mL/min: maximum dose of 150 mg/day, oral.
If needed, it can be increased to 150 mg/day, twice a day, if the GFR is between 30 and 50 mL/min.
19. The patient is receiving a very high dose of allopurinol.
GFR 41-60 mL/min: Maximum dose of 150 mg/day, oral, once a day;
GFR 21-40 mL/min: Maximum dose of 100 mg/day, oral, on alternate days;
GFR 10-20 mL/min: Maximum dose of 100 mg/day, oral, on alternate days;
GFR < 10 mL/min: Maximum dose of 100 mg/day, oral, every three days.
20. The patient is receiving a very high dose of colchicine, as prophylaxis for gout:
GFR < 50 mL/min: chronic treatment not recommended.
The patient is receiving a very high dose of colchicine for an acute treatment:
GFR 35-50 mL/min: maximum dose of 0.6 mg/day;
GFR < 35 mL/min: maximum dose of 0.3 mg, oral, once a day, or 0.6 mg, oral, on alternate days.
21. The patient with a GFR < 30 mL/min is taking a contraindicated medication, a bisphosphonate (alendronate, etidronate, risedronate).
22. The patient is receiving a very high dose of varenicline.
**Inadequate blood pressure**
GFR 10-30 mL/min: maximum dose of 0.5 mg, oral, twice a day;
GFR < 10 mL/min: maximum dose of 0.5 mg, oral, once a day.
Inadequate blood pressure
23. The patient requires medication treatment, because his blood pressure is > 130/80 mmHg, but he is not receiving it.
24. The patient is receiving a very low dose of his anti-hypertensive drug and, consequently, his blood pressure is > 130/80 mmHg.
**Hypoglycemia secondary to sulfonylurea**
25. The patient is developing an adverse reaction (hypoglycemia) after taking his oral hypoglycemia medication (a second-generation sulfonylurea: glibenclamide).
**Interaction and drug taken inadequately**
26. The patient is developing a drug interaction between calcium carbonate and an antibiotic (tetracycline or fluoroquinolone, except moxifloxacin).
27. The patient is developing a drug interaction between calcium and iron taken concomitantly, per os.
28. The patient is not taking his phosphorus scavenger (calcium carbonate, calcium acetate, sevelamer or lanthanum) adequately.
29. The patient is developing a drug interaction between his phosphorus scavenger (calcium carbonate, calcium acetate, sevelamer or lanthanum and levothyroxine).
30. The patient is developing a drug interaction between sevelamer or lanthanum, and ciprofloxacin.
31. The patient is not taking his vitamin D (calcitriol or alfacalcidol) adequately.
32. The patient is not taking his calcium polystyrene sulphonate adequately.
33. The patient needs to the referred to treatment or follow-up on smoke cessation, but has not received it.
**Problems associated with medication not requiring medical prescription or natural health product**
34. The patient is taking medication contraindicated for kidney disease, one antacid with calcium, magnesium, aluminum and/or sodium.
35. The patient is developing an adverse reaction (hypertension) to pseudoephedrine or phenylephrine.
36. The patient is taking a laxative, which is contraindicated for the kidney.
37. The patient is receiving a medication contraindicated for the patient with kidney disease, a polyvitamin enriched with vitamin A.
38. The patient with chronic kidney disease is receiving a very high dose of an ascorbic acid supplement (vitamin C) > 250 mg/day.
39. The patient is receiving a natural product, which is contraindicated in kidney disease, a garlic supplement.
40. The patient is receiving a natural product, contraindicated for transplanted patients with kidney disease, echinacea.
41. The patient is receiving a natural product contraindicated in kidney disease, ginkgo biloba.
42. The patient is receiving a natural product that is contraindicated for transplanted patients, climbing fig (Ficus pumila).
43. The patient is receiving a natural product that is contraindicated in kidney disease, klammath weed (Hypericum perforatum).
44. The patient is receiving a natural product that is contraindicated in kidney disease, liquorice root (Glycyrrhiza glabra).

* Abbreviations: GFR: glomerular filtration rate; DRP: drug-related-product.

The present study aimed to validate the PAIR, assessing its reliability, validity and responsiveness, as well as the prevalence of DRP in a sample of patients with CKD undergoing conservative treatment at a local nephrology outpatient service.

## Methods

This is a longitudinal observational retrospective study conducted in an outpatient nephrology clinic in the city of Juiz de Fora/MG, which is part of a Secondary Health Care center, in which individuals with high cardiovascular risk, SAH, DM and CKD are seen by a multidisciplinary team, including doctors of different specialties, nurses, nutritionists, psychologists, social workers, physical educators and pharmacists.

We analyzed the database of medical records from 100 individuals with a diagnosis of CKD, under conservative treatment, treated between January 2016 and December 2017. For data analysis purposes, we included patients aged 18 years or older in outpatient follow-up for a minimum of 6 months, with data available in medical records. The sample size and the instrument’s validation methodology were reproduced from the original study in English.^10^

To select the medical records, we generated an attendance report (list of patients seen) between the years 2016 and 2017, from the electronic system used in the nephrology outpatient clinic, which contained 4,308 records. From these records, we selected patients who had four consultations at the outpatient clinic during this period, in order to capture those with a confirmed diagnosis of CKD and under regular monitoring by the healthcare team, since, for the analysis we used data from two subsequent consultations of the same patient, with a minimum interval of 6 months. The other inclusion criteria were applied to this list, and 196 patients met these criteria. One-hundred of them were randomly analyzed.

The Research Ethics Committee of the Faculty of Medical and Health Sciences of Juiz de Fora approved the study, according to opinion No. 915,924.

### Sample characteristics

We studied the following sociodemographic, clinical and laboratory variables: ethnics, gender, age, smoking, drinking, schooling, weight, height, body mass index (BMI) according to the BMI formula = weight (kg)/height^2^ (cm), etiology and CKD stage, comorbidities, class and total number of drugs in use, serum creatinine (mg/dl) to estimate the glomerular filtration rate, according to the formula of the Chronic Kidney Disease Epidemiology Collaboration (CKD-EPI).^24^

The cut-off points for BMI adopted were those recommended by the World Health Organization (WHO), that is, low weight (BMI < 18.5); eutrophic (BMI 18.5-24.99); overweight (BMI 25-29.99) and obesity (BMI ≥ 30.00).

### The PAIR measuring instrument

PAIR consists of a list of DRP considered clinically significant for patients with CKD undergoing conservative treatment and requiring pharmaceutical intervention. This list was drawn up using the RAND/UCLA method, developed by a group of researchers from the RAND Corporation and the University of California at Los Angeles (UCLA), which is based on scientific evidence and the agreed opinion of a group of experts, carried out in several phases, for the development of appropriate use criteria in the healthcare field.^10^^,^ 25

The list adapted for Brazil is made up of 44 DRP distributed in 5 categories: 1. Inappropriate use (inappropriate dose or contraindicated medication); 2. Inadequate blood pressure (need for drug treatment or low dose); 3. Hypoglycemia secondary to sulfonylurea; 4. Drug interaction and situations in which the drug is taken inadequately; 5. Problems related to non-prescription drugs or natural health products.^23^

The instrument, in this format, works as a checklist, so that the pharmacist can evaluate the pharmacotherapy of these individuals, identify possible DRP and intervene in their resolution, being important to optimize the clinical results expected by the doctor. This assessment can be made using data provided by the patient himself, in consultation, and/or by data recorded in medical records.^10^

### Data collection

Two independent pharmaceutical examiners, with experience in working at a nephrology outpatient clinic, applied the PAIR instrument by consulting the medical records included in the study, in two stages. First, they evaluated the pharmacotherapy related to the patient’s first consultation after confirming the diagnosis of CKD (pre-intervention period). In the second step, they evaluated data from the same patient in the subsequent consultation, that is, on their return to the clinic after the minimum period of 6 months of follow-up (post-intervention period). For this study, we considered “intervention” to be the specialized follow-up in a multi-professional CKD pre-dialysis service.

For this evaluation, we used the information recorded in the medical records referring to: care of the multidisciplinary team (doctor, nurse, social worker, pharmacist and others); patient’s report on medications in use, including those exempt from prescription, as well as natural products and medical prescriptions from other services unrelated to the outpatient clinic of the study, and results of laboratory tests.

Both examiners received printed material with the appropriate technical guidelines, in addition to specific instructions for data collection and filling out the PAIR.

### DRP Prevalence

The prevalence result was determined by the number of DRP identified by the two pharmaceutical examiners through the PAIR application in 100 patients included in the study, in the pre-intervention. Consensus was used in case of disagreement.

### Reliability

The reliability of the instrument is the ability to reproduce a result consistently in time and space, or from different examiners.^26^

We determined the reliability between examiners by comparing two independent evaluations from the 100 medical records included in the study, in the pre-intervention period. We also used the intra-rater test-retest reliability, which we determined by applying PAIR again, two months after the first assessment, in a random sample of 30 medical records of patients with CKD, using the same data as the pre-intervention.

### Conceptual validity

Validity refers to the ability of an instrument to measure exactly what it is intended to measure. We say that validity is conceptual when it portrays a subjective judgment about the conceptual coverage of an instrument regarding a certain construct.^27^

To achieve this goal, a physician specializing in nephrology evaluated a random sample of 30 medical records of patients with CKD in the pre-intervention, with the goal of detecting clinically significant DRP, based on their implicit clinical judgment, without the aid of the PAIR instrument.

We compared the data obtained with those obtained by the pharmacist, in the same sample, but using PAIR, in order to check whether the instrument was able to detect DRP, which were considered clinically significant by the nephrology specialist.

### Responsiveness

To assess responsiveness, that is, the ability of the instrument to detect clinically important changes over time, 28 the total number of DRP identified by the pharmacist, using PAIR, was computed in the pre-intervention and post-intervention period, as well as the difference between the numbers detected in each period.

Each DRP seen before the intervention was assessed after the intervention for each patient, with the following classification: persistent DRP or resolved DRP (due to a change in pharmacotherapy, results of laboratory tests or treatment compliance). DRP that appeared during the follow-up were also considered in the analysis as new DRP.

### Statistical analysis

We made the descriptive analysis of the data using frequencies, in the case of categorical variables; and means and standard deviations, in the case of quantitative variables.

Considering that patients can have more than one DRP, we determined the prevalence by the proportion of patients presenting with at least one DRP. We also considered the total number of individuals with DRP. We used the Pearson’s correlation coefficient to analyze the association between the variables “number of DRP/patient”, “number of drugs” and “estimated GFR”.

For the PAIR-related DRP, we estimated the reliability between examiners, and for one of them in the retest, using the Cohen’s kappa coefficient (κ), which describes the degree of agreement between the responses. For the purpose of interpreting the κ, we used Landis & Kock’s criteria (1977), who consider that the closer to 1 the value, the greater the indication that there is an agreement.^29^

To assess conceptual validity, we compared the average number of DRP per patient, identified by the pharmacist with the NIHL, with the average number of DRP reported by the nephrologist’s clinical judgment, using the Student’s t-test.

Responsiveness was calculated by means of the average change in the number of DRPs identified between the periods evaluated, using the Student’s t-test to compare the 2 periods.

We adopted 5% as the level of significance, and *p* < 0.05 as statistically significant, using the Statistical Package for the Social Sciences version 17.0 for the Windows software (SPSS Inc., IBM, USA) and the program MedCalc version 19.0.7 (MedCalc Software, Mariakerke, Belgium).

## Results

Among the 100 patients who were eligible for the study, there was a predominance of female individuals (55%), elderly (71%), with an average age of 67 years, with a low level of education - incomplete primary education - (73%) , in stages 3b (38%) and 4 (32%) of CKD, with overweight and obesity (77%), with an average BMI of 30.4 ± 6.1. The most prevalent comorbidities were SAH (96%) and DM (59%). The average use of medications was 7.0 ± 2.7 per patient, considering the number of active ingredients prescribed (Table 1).

**Table 1:** Distribution of CKD patients included in the study according to their demographic and clinical characteristics. Juiz de Fora, 2018 (N = 100)

CHARACTERISTICS	Patientes n = 100 (n/%)	
	N	%
**Gender**		
Female	55	55
Male	45	45
**Age (yrs)**		
≤ 19	0	0
20 - 29	1	1
30 - 39	2	2
40 - 49	6	6
50 - 59	20	20
≥ 60	71	71
**Race**		
White	52	52
Black	23	23
Brown	25	25
**Level of education**		
Can't read/write	10	10
Literate	4	4
Incomplete Elementary school	59	59
Complete Elementary school	13	13
Incomplete High school	3	3
Complete High school	7	7
Incomplete Higher education	1	1
Complete Higher education	3	3
**Smoking**	10	10
**Alcohol abuse**	9	9
**Number of medications**		
2 to 4	22	22
5 to 10	67	67
More than 11	11	11
**CKD stage**		
1	1	1
2	6	6
3a	17	17
3b	38	38
4	32	32
5	6	6
**Body Mass Index (BMI)**		
Underweight	0	0
Healthy weight	21	21
Overweight	29	29
Obesity	48	48
No data	2	2
**Baseline disease**		
Hypertensive nephropathy	23	23
Diabetic nephropathy	23	23
Non-Steroidal anti-Inflammatory Nephropathy	2	2
Undetermined	55	55
**Comorbidities**		
Diabetes	59	59
Hypertension	96	96
Acute Myocardial Infarction	9	9
Cerebrovascular Accident	8	8
**Comorbidities/patient**		
1	22	22
2	40	40
3	28	28
4 or more	10	10

The mean time to apply the NIHL was 9.8 minutes per medical record. We assessed 200 medical prescriptions, 100 in the pre-intervention period and 100 in the post-intervention period. Of these prescriptions, 1,483 drugs with 100 different active ingredients were listed. The most prescribed class of drugs consisted of drugs that act on the cardiovascular system (848 - 57%), followed by drugs with action on the GIT and metabolism (355 - 24%) and on blood and hematopoietic organs (167 - 11%). The most prescribed drugs were losartan potassium (70.5%), simvastatin (62.0%), furosemide (60.0%), acetylsalicylic acid (53.0%), amlodipine besylate (34.0%), omeprazole sodium (30.0%), human insulin NPH (29.5%), metformin hydrochloride (29.5%), atenolol (27.0%), cholecalciferol or vitamin D (23.5%).

### Prevalence of DRPs

In the pre-intervention period, we found 126 DRPs, with an average of 1.26 ± 0.96 DRP per patient. Only 20% of the patients did not have any DRP. The most prevalent categories of DRPs were “interaction and medication taken improperly” (34.1%), “inappropriate use due to inappropriate dose or contraindicated medication” (33.3%), “inadequate blood pressure” (30.2%) (Table 2).

**Table 2:** Prevalence of PAIR DRPs based on consensual evaluation of pharmacotherapy for 100 chronic renal patients under conservative treatment. Juiz de Fora, 2018 (n= 126 DRPs)

Drug Related Problem	Frequency N (%)
**Inadequate use (inappropriate dosage or contra-indicated agent)**	**42 (33.3)**
Non-steroidal anti-inflammatory (DRP 1)	16
Nitrofurantoin (DRP 8)	2
Beta blocker (DRP 14)	2
Fenofibrato. (DRP 15)	3
Metformin (DRP 17)	7
Ranitidine (DRP 18)	4
Allopurinol (DRP 19)	6
Bisphosphonate - alendronate, etidronate, risedronate (DRP 21)	2
**Non-optimal blood pressure**	**38 (30.2)**
Low dose of the antihypertensive agent (DRP 24)	38
**Hypoglycaemia secondary to sulfonylurea**	**1 (0.8)**
Hypoglycaemia after taking glyburide (DRP 25)	1
**Interaction and drug taken inadequately**	**43 (34.1)**
Vitamin D (calcitriol ou alfacalcidol) (DRP 31)	33
No treatment or follow-up on smoking cessation (DRP 33)	10
**Problems related to an over-the-counter medication or a natural health product**	**2 (1.6)**
Purgative not indicated (DRP 36)	2

There was a positive association between the number of drugs and the number of identified DRPs, both in the pre-intervention period (r = 0.221; *p* = 0.02) and in the post-intervention period (r = 0.329; *p* = 0.001). Therefore, the number of DRPs was higher in those patients with a higher number of medications.

**Figure 1:**
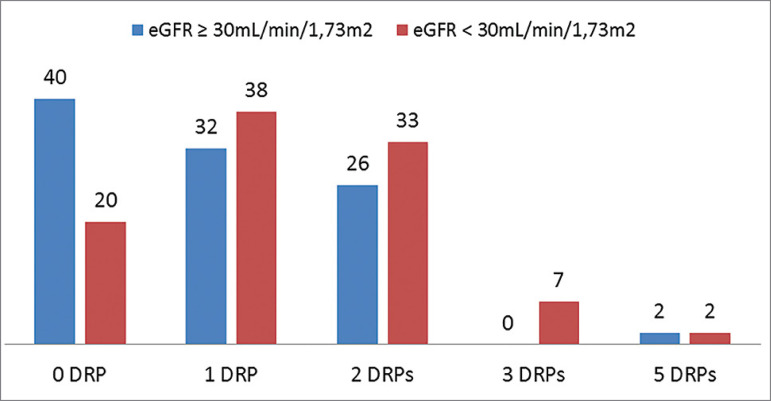
Percentage distribution of the number of DRPs identified per patient according to the estimated glomerular filtration rate. *Chisquared test, *p* = 0.05.

In addition, there was a negative association between the number of DRPs and eGFR in the post-intervention period (r = -0.228 *p* = 0.02), that is, the number of DRPs increased in patients with decreased renal function, defined as eGFR lower than 30 mL/min/1.73 m^2^ (Figure 1).

### Reliability

Considering the total of detected DRPs, the κ coefficient between examiners was 0.45, an indicator of moderate agreement. To detail this analysis, the κ coefficient was also calculated separately for the DRPs representative of the most prevalent categories. In this evaluation, the κ value ranged from 0.58 to 0.94 between examiners, indicating levels of moderate to perfect agreement.

For the test-retest, the κ value for all the DRPs detected in the first and second applications was 0.81. When we assessed the DRPs representative of the most prevalent categories, the κ value ranged from 0.79 to 1.00, indicating substantial to perfect agreement (Table 3).

**Table 3:** Inter-rater and Test-Retest Reliability of the PAIR instrument

Drug Related Problem	Inter-rater Reliability (n=100) κ*	Test-Retest Reliability (n = 30) κ*
**Inadequate use (inappropriate dosage or contra-indicated agent)**		
Nitrofurantoin (DRP 8)	0.662	1.000
Metformin (DRP 17)	0.646	1.000
Allopurinol (DRP 19)	0.790	1.000
**Non-optimal blood pressure**		
Low dose of the antihypertensive agent (DRP 24)	0.723	0.856
**Interaction and drug taken inadequately**		
Vitamin D (calcitriol ou alfacalcidol) (DRP 31)	0.580	0.791
No treatment or follow-up on smoking cessation (DRP 33)	0.942	1.000

* Cohen's *kappa* coefficient.

### Conceptual validity

The nephrologist categorized the DRPs to facilitate their identification in the medical records. According to the doctor’s assessment, the criteria concerning the need to include, suspend or adjust the dose of medications were considered, as clinically significant DRPs.

Based solely on clinical judgment, the physician identified an average of 1.60 ± 1.24 DRP per patient, compared with 1.33 ± 0.76 DRP per patient identified by pharmacists using PAIR, in the same sample (*p* = 0.07).

The medical evaluation resulted in 48 DRPs that required intervention in the prescription, the most frequent being the need for dose adjustment and/or association of antihypertensive drugs (32%) and the need for vitamin D replacement (17%). In comparison, the pharmaceutical evaluation resulted in 40 DRPs, which is in agreement with the medical evaluation, and the most frequent were DRP^24^ “low dose of antihypertensive drug” (28%) and DRP^31^ “vitamin D taken improperly” (33%).

It is worth mentioning some differences between during the medical evaluations when compared with the pharmacists’ evaluations in the identification of some DRPs. The doctor found nine DRPs that were not in the PAIR, related to drugs such as levothyroxine and warfarin. On the other hand, it failed to identify 11 DRPs related to the need to stop smoking and treat the inappropriate use of ranitidine.

### Responsiveness

The average number of DRPS per patient identified by the pharmacist using the NIHL was 1.26 ± 0.96 in the pre-intervention and 1.11 ± 1.02 in the post-intervention (*p* = 0.17). Therefore, it was not possible to detect a difference in the number of DRPs between the periods.

In a more detailed analysis, there were 126 DRPs found in the pre-intervention, 68 DRPs (54%) were resolved and 58 persisted DRPs (46%) found in the subsequent consultation. Thus, in the post-intervention period, out of 111 identified DRPs, 58 persisted and 53 DRP appeared in the follow-up interval.

**Figure 2:**
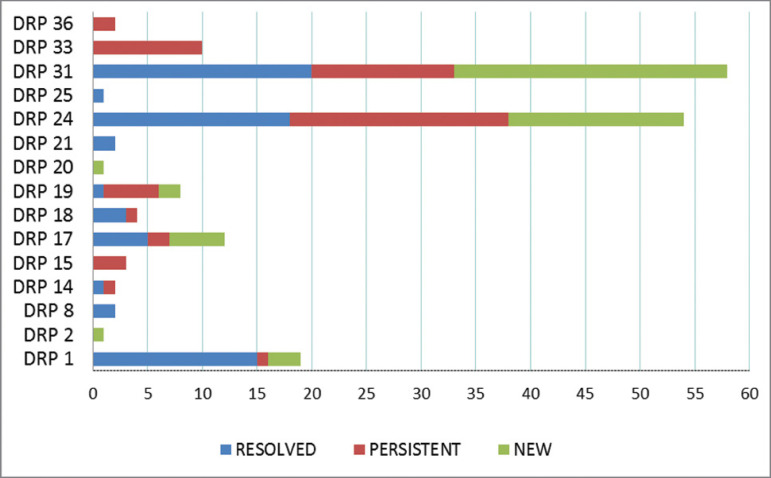
Distribution of DRPs identified during outpatient follow-up, assessed on two occasions within a minimum of six months.

Figure 2 illustrates all the DRPs found during the outpatient’s follow-up and the classification they received when they were reevaluated at the second consultation. In this case, the persistent DRPs (DRPs 1, 14, 15, 17, 18, 19, 24, 31, 33 and 36) deserve to be highlighted, as they failed the intervention.

## Discussion

DRP studies are scarce and are not uniform as to the methodology used for identification and classification, particularly in individuals with CKD under conservative treatment.^7^^,^ 8 ^,^ 30 ^,^ 31 PAIR is an innovative tool, appropriate for the systematic and rapid detection of conditions associated with the safety use of medications that require pharmaceutical intervention, aimed at both the doctor and the patient.^10^ This instrument was recently translated and cross-culturally adapted to Brazilian Portuguese in our service.^23^

The present study aimed to validate the use of PAIR in Brazil. Our results showed that it is an instrument that is easy to apply, and it consumed an average of 9.8 minutes per medical record of patients with CKD undergoing conservative treatment. On average, there were 1.26 ± 0.96 DRP per patient. The most prevalent categories were “drug interactions and medication taken improperly” (34.1%), “inappropriate use due to inappropriate dose or contraindicated medication” (33.3%) and “inadequate blood pressure” (30.2%).

In addition, patients with eGFR lower than 30 mL/min/1.73 m^2^ had a higher number of DRPs. This data is in agreement with the literature, which shows a close relationship between a GFR reduction and a higher number of DRPs.^7^^,^ 8 ^,^ 14 Similarly, patients using multiple medications, a common fact in CKD, are at a higher risk for DRPs. In a study by Kovačević et al (2017), the use of 12 medications was associated with a risk of at least 5 DRPs among the elderly.^32^

Regarding PAIR validation, the reliability found was considered good, with results indicating a moderate to perfect agreement between the DRPs found, both between evaluators and in the test-retest by the same evaluator. These results are similar to those of the validation study of the PAIR`S original, which showed high reliability between evaluators, with k coefficients ranging from 0.80 to 1.00, and high reliability in the test-retest, with k coefficients ranging from 0, 74 to 1.00.^10^ Other pharmacotherapy assessment instruments based on specific criteria, such as some designed for use in the elderly, the Screening Tool of Older Person’s Prescriptions (STOPP) and the Screening Tool to Alert doctors to Right Treatment (START), showed values k among evaluators equal to 0.75 and 0.68, respectively,^33^ similar to the values ​​found in the present study.

Specific to this study, the kappa values obtained can be attributed to the study design, and because we used data from electronic medical records for data collection. Although the medical records investigated are complete, certain information, subjective to clinical practice, was recorded in different fields on the website, given the variation of professionals responsible for care, a fact that probably generated differences in data collection between the examiners. Thus, there was a weakness in the electronic service system used in the outpatient clinic under study, since it is an internal system, designed especially for that clinic. In the years 2016 and 2017, such system was in a constant process of change, including changes to its layout. However, regardless of the overall calculated kappa value, the prevalence rates for each separate DRP, for each examiner, were similar in percentage and involved the same patients.

Regarding conceptual validity, our results revealed that the PAIR was able to identify clinically significant DRPs in patients with CKD, using the implicit clinical judgment of a nephrologist as a parameter for this analysis. Based solely on clinical judgment, the nephrologist found the number of DRPs per patient comparable to the number identified by the pharmacist, with the aid of the PAIR instrument in the same sample. The differences found can be attributed to the specificity of the instrument, since the doctor made a general analysis of the patient’s health condition aimed at his clinical improvement and the pharmacist performed the search for DRPs following a predetermined checklist. This finding is similar to the comparative analysis performed by Desrochers et al, in which the nephrology specialist clinically identified an average of 3.9 (95% CI, 3.4-4.5) DRPs per patient and the community pharmacist, using the PAIR, found an average of 2.5 (95% CI, 2.0-3.1) DRPs per patient. In this study, PAIR allowed managing DRPs with an important clinical impact in the treatment of CKD.^10^ Therefore; our data show that the PAIR instrument is suitable for the management of clinically significant DRPs in Brazilian Portuguese.

However, regarding responsiveness, our results do not suggest that PAIR is responsive to pharmaceutical intervention, since the average number of DRPs per patient remained stable between the pre and post-intervention periods. In the meantime, only 54% of DRPs were resolved due to the optimization of medical prescription. In fact, we expected a decrease in the number of DRPs in the post-intervention period. However, this study was retrospective and observational, and, during the follow-up period at the outpatient clinic, the patient was subjected to routine interventions, with no pharmaceutical interventions that specifically contemplated the DRPs present in the PAIR. In addition, the interval between periods was short for this type of analysis.

These same reasons were claimed by the researchers in the original study carried out in Canada, which also found no difference in the mean number of DRPs detected with the PAIR instrument in the six-month evaluation period. The mean number of DRPs remained equal to 2.5 per patient, with only 61% of DRPs being resolved in the interval between periods.^10^ Thus; it was not possible to assess the PAIR, due to the protocol’s characteristic, which reinforces the need for prospective and multicenter studies.

Given the above, it is necessary to consider some limitations to the present study. PAIR has checklist characteristics, that is, it works as a security instrument, as it consists of a set of conducts that must be remembered and/or followed in order to avoid and/or detect DRPs. 

Thus, PAIR’s applicability is related to the characteristics of the service provided to the user from a clinic that clearly contemplates these conducts as an institutional protocol, so that the DRPs are not underestimated and the interventions can be effective. In addition, in practice, there may be a need to update the PAIR’s list of DRPs, considering the time it was developed, the dynamics of the pharmaceutical industry and the incorporation of new technologies and healthcare evidence, which require changes to clinical guidelines.

However, the limitations described above do not compromise PAIR’s use in the population of patients with CKD in our country. On the contrary, they contribute so that information regarding the pharmacotherapy of this population is not neglected, since this was the first study developed in Brazil with the aim of validating an instrument aimed at the safety of medication in CKD.

## Conclusion

The present study demonstrated that PAIR is easy to apply, reliable, and its use has been validated for Brazil. It is, therefore, an adequate instrument for the evaluation of clinically significant DRPs in our population of patients with CKD.

The incorporation of this instrument in pharmaceutical care in nephrology services may allow for the systematization and standardization of data, thus enabling the implementation of prevention and management strategies for frequent DRPs in this population.
